# Hybrid cell reactor system from *Escherichia coli* protoplast cells and arrayed lipid bilayer chamber device

**DOI:** 10.1038/s41598-018-30231-0

**Published:** 2018-08-06

**Authors:** Yoshiki Moriizumi, Kazuhito V. Tabata, Rikiya Watanabe, Tomohiro Doura, Mako Kamiya, Yasuteru Urano, Hiroyuki Noji

**Affiliations:** 1 Department of Applied Chemistry, Graduate School of Engineering, The University of Tokyo, 7-3-1, Hongo, Bunkyo-ku, Tokyo, 113-8656 Japan; 2 Graduate School of Medicine, The University of Tokyo, 7-3-1, Hongo, Bunkyo-ku, Tokyo, 113-0033 Japan; 3 Graduate School of Pharmaceutical Sciences, The University of Tokyo, 7-3-1, Hongo, Bunkyo-ku, Tokyo, 113-0033 Japan; 4 PRESTO, Japan Science and Technology Agency, 4-1-8, Honcho, Kawaguchi, Saitama, 332-0012 Japan; 5 PRIME, Agency for Medical Research and Development, 1-7-1, Otemachi, Chiyoda-ku, Tokyo, 100-0004 Japan; 6 CREST, Agency for Medical Research and Development, 1-7-1, Otemachi, Chiyoda-ku, Tokyo, 100-0004 Japan; 7 ImPACT, Council for Science, Technology and Innovation, Cabinet office, Government of Japan, 1-6-1, Nagata-cho, Chiyoda-ku, Tokyo, 100-8914 Japan

## Abstract

We developed a novel hybrid cell reactor system via functional fusion of single *Escherichia coli* protoplast cells, that are deficient in cell wall and expose plasma membrane, with arrayed lipid bilayer chambers on a device in order to incorporate the full set of cytosolic and membrane constituents into the artificial chambers. We investigated gene expression activity to represent the viability of the hybrid cell reactors: over 20% of hybrid cells showed gene expression activity from plasmid or mRNA. This suggests that the hybrid cell reactors retained fundamental activity of genetic information transduction. To expand the applicability of the hybrid cell reactors, we also developed the *E*. *coli*-in-*E*. *coli* cytoplasm system as an artificial parasitism system. Over 30% of encapsulated *E*. *coli* cells exhibited normal cell division, showing that hybrid cells can accommodate and cultivate living cells. This novel artificial cell reactor technology would enable unique approaches for synthetic cell researches such as reconstruction of living cell, artificial parasitism/symbiosis system, or physical simulation to test functionality of synthetic genome.

## Introduction

Louis Pasteur proposed the biological dogma, which states that living organisms can only be generated from living organisms, and this dogma has yet to be overturned. Reconstruction of a living cell from inanimate molecules represent one of the ultimate challenges in biology. To reconstruct a living cell, one must reconstitute essential cellular functions for the self-replication of genetic materials, expression of genetic information, energy transduction, and biosynthesis of constituents. Encapsulation of molecular components in a small compartment is also required for cell reconstruction^1^ to set physical boundaries that prevent invasion of non-self genetic materials such as parasites and allows for Darwinian evolution^2,3^.

Many attempts at developing artificial cells have been reported, in which a cell function(s) is reconstituted in a micro-compartment^1,4–7^. In most studies, a gene expression system composed of transcription and translation components^8,9^ is implemented as an *in vitro* transcription-translation (TX-TL) system in artificial cells because of the physiological importance and application potential of these systems^10^. A self-replication system of genetic materials, including DNA or RNA, has also been implemented into micro-compartments^2,11–13^. *In vitro* reconstitution of genome replication of *Escherichia coli* has recently been reported^14^. These techniques have provided a foundation for artificial cell research. Particularly, full reconstitution of the central reactions constituting the central dogma has become experimentally accessible. The reconstitution of such integrated systems in micro-compartments is a step towards full reconstitution of an autonomous self-replication system *in vitro*.

For micro-compartmentalization, vesicles composed of phospholipid, liposome, are widely used^15–18^ because liposomes offer a physical boundary very similar to natural cell membranes and allow for reconstitution of functional membrane proteins^19,20^. Fatty acid-based vesicles have been evaluated as another model in protocell studies^21,22^. These vesicles also allow for passive diffusion of small molecules across the membrane, enabling uptake of nutrient molecules^23^. Water-in-oil (W/O) droplets are also widely used in artificial cell research because of the high controllability of size and ease of preparation that allows for high-throughput production^24^. Because of these advantageous features, quantitative analysis in cell-free assays, such as TX-TL or gene circuits, are performed inside of W/O droplets^2,25–28^. Microreactors or microchannels on microsystems have attracted attentions as alternatives to soft material-based microcompartments. Microreactors integrated with DNA chip technology for cell-free gene expression in a finely controlled manner were reported^29,30^.

Thus, artificial cell studies have successfully expanded variations and applications, enabling multiplex or multistep reactions such as the cascading genetic network^31^, self-oscillating system^25,28,30^, and bacteria phage reconstitution^32^. However, it remains extremely difficult to reconstruct an autonomously self-replicating system at least due to the practical difficulty to reconstitute a full set of constituents from purified components. Thus, it would be worth to ask the possibility to reproduce a ‘living’ cell simply by the incorporating a full set of constituents from a single living cell into an artificial microcompartment. Pioneering studies have attempted reconstitution of a living cell from cell extract^15,16,33^. Although these studies detected translation activities, the reconstituted artificial cells did not have potentiality for self-replication due to the lack of functional membranes. Additionally, the cell extracts were substantially diluted during preparation from highly condensed cytoplasm^34,35^. To retain the viability, the cell extract dilution should be minimized to avoid an irreversible loss of viability^36,37^.

In the present study, we incorporated a full set of constituents from a single living *E*. *coli* cell into an artificial cell reactor system known as an arrayed lipid bilayer chamber system (ALBiC)^38^. Each reactor of the ALBiC has a volume of 25 fL. Because the upper aperture of the reactors is sealed with lipid bilayer, the ALBiC allows for incorporation of membrane proteins via membrane fusion. In this study, we prepared protoplast cells of *E*. *coli* by repelling the outer membrane and most of the peptide glycan layer for efficient membrane fusion^39^. *E*. *coli* protoplasts were placed onto the reactors and showed spontaneous membrane fusion with the lipid bilayer of the ALBiC. Thus, all components were introduced into the inner space of the ALBiC reactors. We named the fused reactor as a “hybrid cell”. We measured the protein synthesis activity of the hybrid cell as an indicator of cell viability. To expand the applicability of the hybrid cell reactors and to investigate possible intracellular interplay between the hybrid cell and living cells, we developed *E*. *coli*-in-*E*. *coli* cytoplasm, in which entrapped *E*. *coli* cells showed normal cell division as a novel platform of an artificial parasitism system and cellular bionics system^40^.

## Results

### Artificial cell reactor, ALBiC

**Figure 1:**
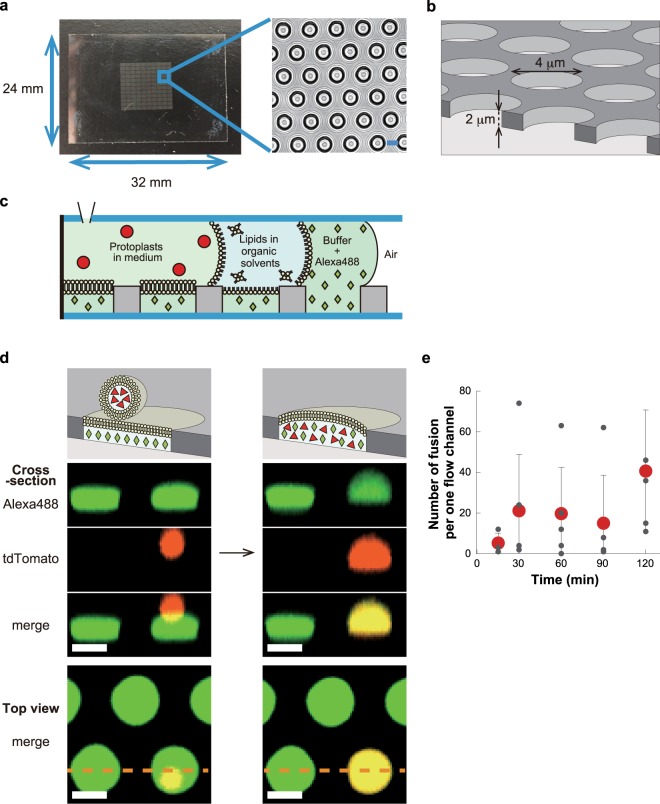
Hybrid cells made from *E*. *coli* protoplast cell and arrayed lipid bilayer chamber (ALBiC). (**a**) ALBiC device viewed from top. The enlarged image is a bright field image of chambers. Scale bars are 4 μm. (**b**) Schematic illustration of ALBiC device. The diameter and depth of each reactor are 4 and 2 μm, respectively, and resultant volume is 25 femtoliter (=25 × 10^−15^ *L*). In this study, other devices with different size (4.5 μm in diameter and 3 μm in depth) were also used. (**c**) Schematic illustration of lipid bilayer formation viewed from side. A flow channel was composed of an ALBiC device and top cover glass with a spacer sheet. Media were sequentially introduced into a flow chamber from a hole in the top cover glass. After the lipid bilayer was formed on the top orifice of the chambers, protoplast cells were introduced with a culture medium. (**d**) Fusion process of protoplast cell. Schematic illustration (top) and experimentally recorded images (lowers). Alexa488 (green diamonds) emitting green fluorescence was encapsulated in ALBiC reactors to confirm that lipid bilayers were maintained. Red florescence represents *E*. *coli* protoplast cells expressing tdTomato protein (red triangles). When a protoplast cell was fused with an ALBiC reactor, red fluorescence spread throughout the reactor as shown. Cross-section images correspond to the side views of the reactors at the orange dotted line in the top views (bottom). Colocalization of red and green fluorescence confirmed mixing of the protoplast cytosol and the solution in an ALBiC reactor (indicated as a yellow signal in merge images). Upon the fusion, the total volume of hybrid cells increased and the hybrid cells resultantly formed a hemispherical shape. Scale bars are 4 μm. (**e**) Time course of fusion per flow channel. Time 0 was defined as the time of protoplast medium introduction into a flow channel. Gray and red dots indicate the number of fusion event in individual experiments and the averaged ones. Experiments were repeated 3–5 times. Error bars represent standard deviation.

ALBiC devices (Fig. 1a,b) were prepared as described previously^38^. A single ALBiC device has one million micron-sized holes of fluorinated polymer layer that was cast on a glass coverslip. The micron-sized holes were used as reactors. Flow cells having two flow channels were formed from an ALBiC device and a top coverslip, between which a spacer was inserted. Two flow channels, each with 100–200 thousand reactors, were used for independent experiments. Lipid bilayers were formed on the upper aperture of the rectors (Fig. 1c). First, an aqueous solution was injected into the flow channel to fill the reactors. Next, hexadecane containing lipid molecules were injected. Excess aqueous solution was flushed, and a ‘mono’-layer of lipid was formed at the water/oil interface at the reactor apertures. A secondary aqueous solution was introduced to flush the organic solvent to form the secondary monolayer at the second water/oil interface. When the second monolayer ran over the orifice, the two monolayers were sealed to form a bilayer sheet. To monitor the integrity of the lipid bilayer during experiments, a membrane-impermeable fluorescent dye, Alexa Fluor 488 (Alexa488) or Alexa Fluor 405 (Alexa405) was entrapped in the reactors with the first aqueous solution. When the bilayers ruptured, fluorescence disappeared, enabling identification of the position of ruptured reactors.

### Fusion of *E. coli* protoplast to ALBiC reactor

To enhance the fusion of *E*. *coli* cells to the ALBiC, *E*. *coli* cells were treated with lysozyme for cell wall removal as previously reported^39^. Resultant round-shaped *E*. *coli* protoplast cells had an exposed plasma membrane, allowing direct contact of the plasma membrane with the ALBiC bilayer. Because of the higher density of protoplast cells compared to the medium, protoplast cells were spontaneously sedimented, and some of them landed on the lipid bilayers of the ALBiC (Fig. 1d). Membrane fusion of protoplast cells and ALBiC stochastically occurred and was monitored by red fluorescent signal of tdTomato expressed in the protoplast cells (Fig. 1d). Upon membrane fusion, red fluorescence from a protoplast cell disappeared, and at the same time red fluorescence spread in the reactor beneath the protoplast. Cross-section images of formed hybrid cells showed co-localization of red and green signals (Fig. 1d), confirming that cytoplasmic constituents were mixed with the inner solution of the reactor, while the integrity of the ALBiC lipid bilayer was retained.

We observed 20–100 fusion events per flow channel with approximately 160,000 reactors. To investigate the kinetics of the fusion, we measured the number of fusion events at different incubation times (Fig. 1e). In each assay, protoplast cells floating in the flow channel were washed out at indicated times in Fig. 1e, and the total number of fused reactors was counted. Hybrid cells were identified as the reactors filled with the red fluorescence of tdTomato. As shown in Fig. 1e, fusion was nearly complete within the first 30 min, and did not subsequently increase. The total number of hybrid cells plateaued at approximately 40 cells per flow channel. The fraction of protoplast cells that fused with the reactors was as low as 0.3% of the total cells that landed on the ALBiC lipid bilayer. This suggests that the fusion is highly conditional; only a very small fraction of protoplasts fused. Some microscopic features such as chemical compositions or membrane structures may trigger membrane fusion. Because of the low fusion efficiency, two or more successive fusions per one reactor were not observed.

### Incorporation of membrane components into ALBiC reactor

**Figure 2:**
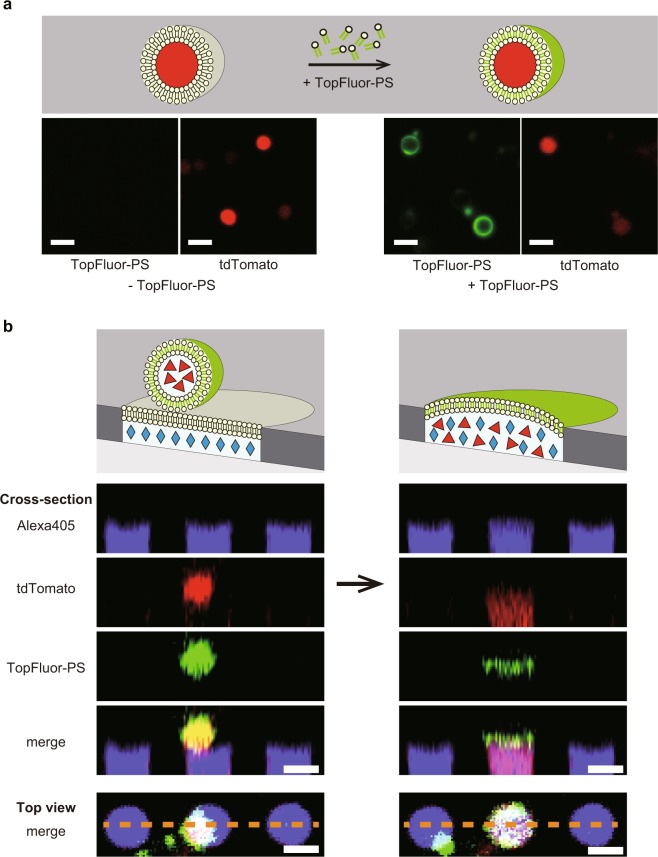
Fusion of membrane components of protoplast cells with ALBiC. (**a**) Staining of the protoplast membrane with the fluorescent-labeled lipid, TopFluor-PS. Schematic illustration (upper) and experimentally recorded images without and with the addition of TopFluor-PS (lower). (**b**) Fusion of the protoplast of fluorescent-labeled membrane. Schematic illustration (top) and experimentally captured images (lowers). Cross-section of top views (bottom) along the orange dotted lines. Alexa405 emitting cyan fluorescence (cyan diamonds) was encapsulated in ALBiC reactors. Red florescence represents *E*. *coli* protoplast cells expressing tdTomato protein (red triangles). The green signal from protoplast resulted from the membrane staining with TopFluor-PS. After fusion, red and cyan fluorescence signal colocalized in the reactor (indicated as a pink signal in merge images). Besides, green signal of TopFluor-PS localizes at the orifice of the reactor, showing fusion of the plasma membrane of protoplast and the lipid bilayer of ALBiC. Scale bars are 4 μm.

One of the distinctive features of the hybrid cells that discriminate it from previous artificial cells technology is the incorporation of the full set of membrane components into artificial cell lipid bilayer. To visualize the membrane fusion, we stained protoplast with fluorescent lipid, 1-palmitoyl-2-(dipyrrometheneboron difluoride)undecanoyl-*sn*-glycero-3-phospho-L-serine (TopFluor-PS)^41,42^ (Fig. 2a) and fused with ALBiC (Fig. 2b). To suppress background fluorescence of ALBiC bilayer due to lipid aggregates stained with TopFluor-PS, protoplast solution was extensively diluted, over 50-hold, from the above condition. Although the number of the fusion events were largely reduced, some hybrid cells were identified. As shown in Fig. 2b, the fluorescent signal from TopFluor-PS originated from stained protoplast cells was observed at the position of the lipid bilayer of hybrid cells. It is evident that fluorescent membrane of protoplast was fused with lipid bilayer of ALBiC. Thus, it was confirmed that hybrid cells possess membrane constituents from a single living protoplast cell in addition to cytoplasmic components.

To confirm the functionality of membrane proteins incorporated into the ALBiC lipid bilayer, the proton transport activity of cytochrome *c* oxidase, which is known as a highly active proton pump, was measured by use of a voltage sensitive dye, tetramethylrhodamine (TMRM). The reaction was initiated by adding ubiquinol-1, a membrane-permeable quinone. However, obvious activity was not observed because of nonspecific incorporation of the dye onto the device surfaces that hampered the analysis.

### Cultivation of hybrid cell

One of the most fundamental nature of living cells is self-replication which is always accompanied with active growth and deformation activity of membrane as seen in expansion, protrusion, and fission of cells. We first examined the membrane dynamics of hybrid cells as a sign of self-replicating activity, expecting membrane budding as seen in the L-form bacteria^43–46^ that shows active membrane expansion and fissions without any molecular machinery for cell division like FtsZ^47^. It is also known that vesicles can show large membrane budding or protrusion driven by so-called excluded volume effect by under high surface-to-volume ratio conditions^48,49^. We cultivated hybrid cells on a microscopic stage for 15–20 h. The total observation time was determined, taking account of the observations that 23% of intact protoplast cells showed significant membrane morphological changes such as expansion, protrusion, or division within 15 h cultivation and that 80–85% of them showed such a morphological change within 3 h. We observed totally 434 hybrid cells. However, hybrid cells did not show clear morphological deformation of membrane. This means that hybrid cells did not retain active membrane growth and deformation activity even equipped with membrane components from living cell. It should be noted that only when the inner solution of chambers or lipid bilayer of chambers was not stained with fluorescent dyes, we observed that vesicle structures with fluorescent signal of GFPuv embedded in the cavity of the ALBiC device showed large morphological changes of their membranes; expansion, and protrusion from the device (Supplementary Movies 1 and 2). However, without staining dyes, it is not clear whether the vesicle structures showing morphological changes were hybrid cells or intact protoplast cells held in the empty chamber after spontaneous rupture of lipid bilayer.

### Gene expression from plasmid DNA entrapped in hybrid cell

**Figure 3:**
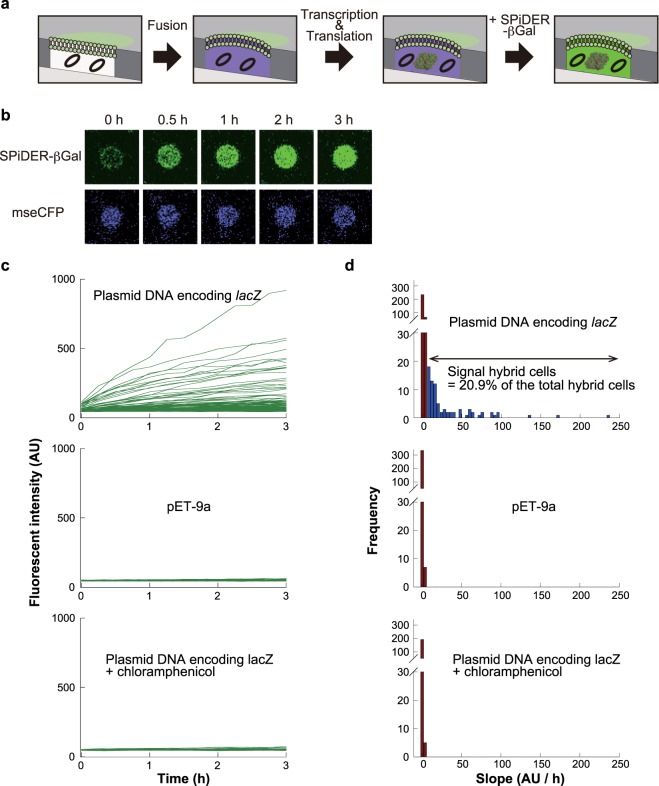
Gene expression of hybrid cells from encapsulated plasmid DNA. (**a**) Schematic illustration of the assay. Black circles indicate plasmid DNA molecules encoding β-gal gene. After the fusion of protoplasts expressing mseCFP and following incubation for 3 h, a membrane-permeable fluorogenic substrates, SPiDER-βGal, was introduced. Upon cleavage by β-gal enzyme, SPiDER-βGal is transformed to membrane-impermeable compound and accumulated inside hybrid cells. (**b**) Fluorescence images of an active hybrid cell including after SPiDER-βGal injection. Time-lapsed images of SPiDER-βGal fluorescence (upper) and mseCFP fluorescence (lower) images from the same hybrid cell. (**c**) SPiDER-βGal fluorescence time courses of hybrid cells. Hybrid cells including plasmids encoding β-gal gene (*lacZ*) (upper), empty plasmids (pET-9a) (middle) and *lacZ* plasmid with chloramphenicol (lower). Each line indicates one hybrid cell. (**d**) Histograms of fluorescence increment rates. The upper histogram shows the rates of hybrid cells carrying plasmid encoding β-gal gene. The middle and lower histograms represent data of control experiments: using pET-9a and chloramphenicol. Deep red bins indicate inactive chambers defined from the histograms of control experiments. The proportion of hybrid cells active in the gene expression of β-gal was 20.9% (blue bins).

We further tested the viability of hybrid cells, by evaluating the gene expression activity of hybrid cells. To monitor the activity with high sensitivity, we measured enzyme expression from plasmid DNA molecules entrapped in ALBiC reactors (Fig. 3a). For this purpose, we prepared ALBiC reactors, each entrapping 2.73 × 10^2^ copies of purified plasmid DNA molecules encoding *lacZ*, the gene for β-galactosidase (β-gal), for which a highly sensitive fluorogenic assay is available^50,51^. *lacZ* was encoded under the *lac* promoter and a protoplast cell from a *ΔlacI* strain was used. Thus, the resultant hybrid cell was designed to express β-gal without induction. In this experiment, we used protoplasts expressing cyan fluorescent protein, mseCFP, to avoid optical overlap with fluorogenic assay of β-gal. 5 mM adenosine triphosphate (ATP) was added to the ALBiC reactors with plasmid DNA, considering preliminary experiments that showed 5 mM ATP enhanced gene expression activity while other compounds such as amino acids or glutathione did not.

To detect β-gal, the fluorogenic substrate Spiro-based immobilisable diethylrhodol-βGal (SPiDER-βGal) was used instead of Fluorescein di-β-D-galactopyranoside (FDG) that is commonly used as a fluorogenic substrate in β-gal assays. This is because the FDG is not suitable for hybrid cells, as fluorescein, the hydrolyzed product of FDG by β-gal, is a small molecule and is relatively easily leaked out from the hybrid cells (Supplementary Fig. 1). SPiDER-βGal is a membrane-permeable compound that is readily introduced into cells when added to the external solution^52^. Once cleaved by β-gal enzyme, SPiDER-βGal transforms into a membrane-impermeable compound by forming a fluorescent conjugate with macromolecules such as proteins. To avoid possible interference of gene expression activity by SPiDER-βGal, a solution containing SPiDER-βGal was injected into the flow channel after 3 h of incubation of hybrid cells. Some hybrid cells showed a clear green fluorescent signal (Fig. 3b,c). When an empty plasmid, pET-9a (*ΔlacZ*), was introduced for comparison, a fluorescence signal was not observed in any hybrid cells (Fig. 3c). When hybrid cells were treated with chloramphenicol, which inhibits gene expression, a fluorescence signal was not observed. Thus, green fluorescence represents the actual gene expression activity in the hybrid cells.

Figure 3d shows histograms of gene expression activity of hybrid cells, with control experimental data: hybrid cells with empty plasmid and hybrid cells treated with chloramphenicol. Control experimental data set the upper limit of false-positive signals of gene expression activity at 8.0 AU/h. Thus, we defined active hybrid cells in gene expression as those with signals greater than 8.0 AU/h. Under our experimental conditions, the fraction of active hybrid cells was 20.9%. To estimate the number of β-gal molecules expressed in the hybrid cells, we analyzed the catalytic activity of purified β-gal in ALBiC reactors at different numbers of β-gal molecules per reactor (Supplementary Fig. 2a). The mean number of expressed β-gal molecules in hybrid cells was estimated to be 25 molecules (Supplementary Fig. 2b), although the numbers were largely different among hybrid cells, showing a Poisson-like distribution. We also observed the gene expression of β-gal from plasmid DNA in intact protoplast cells for comparison purpose. Cells showed evidently higher enzyme activity, than hybrid cells. Although the copy number of plasmid DNA in cells and other experimental conditions were different from hybrid cells, this suggests that the gene expression activity of active hybrid cells was significantly lower than intact protoplast cells.

We also investigated the correlation between gene expression activity and the dilution rate of protoplast cytosol for individual fusion events. We estimated the dilution rate of protoplast cytoplasm from the intensity decrease in Alexa405 fluorescence, which was added to the inner solution. The dilution rate ranged from approximately 1.0 to more than 20 with a mean of 3.0 (SD: 5.89). Supplementary Fig. 3 shows the histogram of the population of active hybrid cells in gene expression against the dilution rate. While the number for highly diluted hybrid cells was statistically too small to judge the correlation, the data points for low dilution rates (1.0–2.0, 2.0–3.0, and 3.0–4.0) showed that the population of active hybrid cells was principally constant. This suggests that other factors such as the original viability of the protoplast cells determine the gene expression activity of hybrid cells.

### Gene expression from mRNA entrapped in hybrid cells

**Figure 4:**
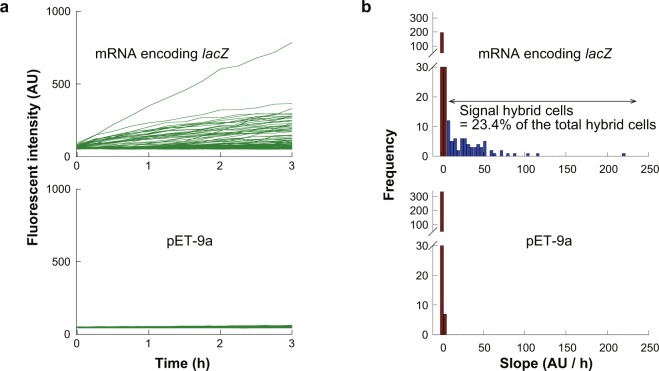
Gene expression of hybrid cells from encapsulated mRNA. (**a**) Time courses of gene expression from *lacZ*-coding mRNA molecules encapsulated in ALBiC reactors (upper). Expression activity was monitored with SPiDER-βGal fluorescence as shown in Fig. 3. Control experiment using pET-9a (Fig. 3c) was also shown for comparison. (**b**) Histograms of fluorescence increment rate of hybrid cells. The upper shows the data from hybrid cells carrying *lacZ*-coding mRNA. The lower shows data of the control experiment (Fig. 3d). Red bins indicate inactive chambers defined from the histograms of the control experiment. Gray bins indicate the number of hybrid cells with no β-gal signal, while blue bins indicate the number of hybrid cells with β-gal signal. The total number of hybrid cells active for expression of β-gal signal was 23.4%.

We also tested the gene expression activity from mRNA. mRNA molecules encoding *lacZ* were encapsulated in ALBiC reactors prior to fusion of protoplast cells. To maximize gene expression, excess mRNA 1.44 × 10^3^ mRNA molecules per reactor) was introduced. The number of enclosed mRNA was comparable to the total transcripts per *E*. *coli* cell, on the order of 1000^53^. Gene expression was measured by adding SPiDER-βGal after 3 h of incubation of hybrid cells (Fig. 4a). The fluorescence signal of β-gal catalysis was analyzed as described above (Fig. 3). The fraction of active hybrid cells was determined to be 23.4% (Fig. 4b). Although this value is slightly higher than that for expression from DNA, the fraction of active cells was not greatly improved. The fluorescence signal of active cells mostly ranged from 10 to 100 AU/h, which did not differ from the gene expression from plasmid DNA. These observations suggest that the bottleneck of gene expression is translation.

### Entrapment of living *E. coli* cell in hybrid cells

**Figure 5:**
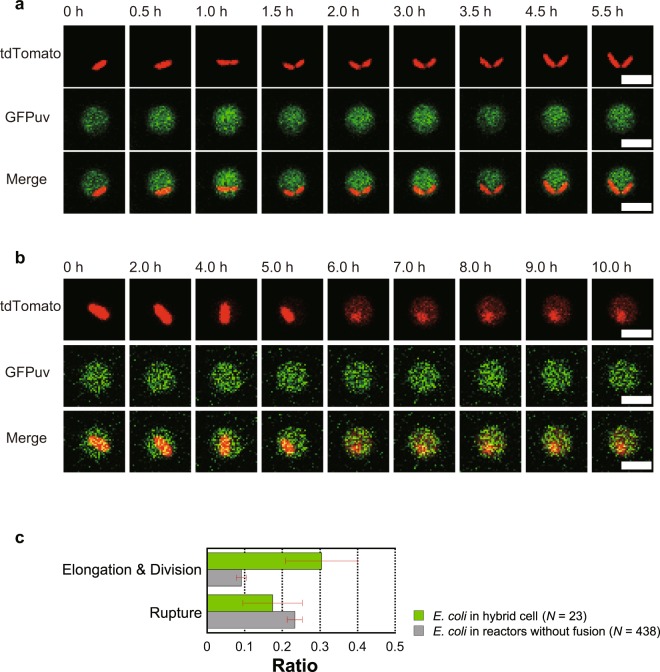
Cultivation of *E*. *coli* cells in hybrid cells. A tdTomato-expressing *E*. *coli* cell (red) was encapsulated in a hybrid cell formed with GFPuv-expressing protoplasts (green). Encapsulated *E*. *coli* cells showed active cell division (**a**) or cell lysis (**b**). Scale bars indicate 4 μm. (**c**) Ratios of cells showing active elongation/division or cell lysis. For comparison, corresponding ratios of cells in ALBiC reactors that did not fuse with a protoplast cells are shown as gray bars. Error bars indicate s.e.

The above experiments demonstrated the functional encapsulation of macromolecules such as plasmid DNA and mRNA with molecular weights of 100–1000 kDa. Here, to test the potential for encapsulating larger and more complex systems inside the hybrid cells, we attempted to encapsulate living *E*. *coli* cells in hybrid cells. For this purpose, we prepared an ALBiC reactor entrapping living *E*. *coli* cells expressing tdTomato. By fusing protoplast cells expressing GFPuv to the ALBiC reactors, we prepared hybrid cells containing a living bacterial cell (Fig. 5). When incubated at 30 °C for 4–6 h, some entrapped *E*. *coli* cells elongated and divided into two cells (Fig. 5a). The fraction of cells showing cell division in the hybrid cells (30.4%) was significantly higher than the fraction of cells entrapped in the ALBiC reactor that was not fused with protoplast cells (9.1%) (Fig. 5c). This suggests that cytosolic components of hybrid cells supplied molecular materials and energy for entrapped cells to produce biomass, leading to cell division. Although the inner medium of ALBiC reactors did not contain nutrient components at the beginning of the experiments, 9% of entrapped *E*. *coli* cells showed cell division. This is attributable to the passive diffusion of nutrient molecules into ALBiC reactors from flow channel containing culture medium. Interestingly, some entrapped *E*. *coli* cells (17.4%) ruptured, spreading tdTomato fluorescence in the reactor (Fig. 5b). The rupture was also observed in empty reactors (23.3%). These results suggest that the physical interaction with device surface induced bacterial lysis.

## Discussion

We developed a novel method for building an array of hybrid cells from single living bacterial cells and artificial cell reactors displayed on an ALBiC device^38^. This was accomplished using the advantageous features of the ALBiC; this device is composed of solid state materials (glass and a fluorinated polymer sheet) and a representative of soft materials. The solid-state materials principally defined the size and shape of the hybrid cell reactors. The regularly shaped ALBiC reactors allowed for quantitative analysis of gene expression activities in the hybrid cells. The detection and quantitative analysis of minor fractions of hybrid cells were also allowed based on the parallelism of ALBiC, *i*.*e*. the device displayed almost a million reactors on its surface. Thus, minor groups of hybrid cells showing interesting features were identified for analysis. The lipid bilayer formed on the orifice of the ALBiC reactor functioned as the dynamic interface between the interior and exterior spaces of the hybrid cells. This dynamic interface enabled incorporation of a full set of components from single living *E*. *coli* cells into reactors via membrane fusion. Upon the membrane fusion, the cell plasma membrane components including membrane proteins like transporters, and receptors were incorporated in the lipid bilayer to form a hybrid cell membrane. This discriminates the present study from other artificial cell studies in which none or a few kinds of membrane proteins were reconstituted into vesicles.

For efficient incorporation of all components from a single living cell, protoplast cells of *E*. *coli* were prepared by removing the outer membrane and the cell wall mainly composed of peptidoglycans^39^. The protoplast exposing the plasma membrane fused with the ALBiC reactor via membrane fusion (Fig. 1d). The fraction of protoplast cells that fused with the reactors was low at only 0.3% of the total cells landing on the ALBiC lipid bilayer. Thus, the yield of hybrid cells in the present protocol was low. However, this is reasonable considering that liposome-liposome or liposome-planner bilayer fusion generally does not frequently occur unless using a chemical or electrical stimulus^54,55^. Several studies showed that in the absence of fusion enhancing reagents or stimulus, the liposome-liposome fusion does not occur or even at the best cases several times per 100 μm^2^ ^56–60^. The fusion rate of protoplast into ALBiC bilayer corresponds to 1.5~2.3 × 10^−2^ times per 100 μm^2^ within the reported values. Protoplast-protoplast fusion is even less frequent, in the best case 0.07%^61^, probably because densely crowding membrane proteins hamper the membrane fusion. Protoplast-liposome fusion that is more closed to the present study condition has not been quantitatively analyzed yet, but would be more frequent than protoplast-protoplast fusion. Thus, the fusion rate observed in the present work is reasonable. In order to enhance the fusion efficiency, we tested chemicals that induce membrane fusion such as polyethylene glycol (PEG) in preliminary experiments. However, these chemicals often weakened the durability of the lipid bilayer of the ALBiC reactors and did not improve fusion yield. Membrane fusion-assisting proteins such as SNARE proteins may enhance fusion^62^.

We expected the growth activity of hybrid cell, considering L-form bacteria^43–46^ which showed active membrane deformation and cell division under the conditions when cell wall synthesis was completely abolished and osmotic pressure of the medium is balanced with that of cells. L-form bacterial cells, thus, can divide their cell body independently of the FtsZ system. The L-form is considered a special form of protoplast cells. Thereby, it would be reasonable to expect the hybrid cells also show active membrane expansion and membrane protrusion. However, observed hybrid cells did not show active membrane growth during 15–20 h cultivation. Recent studies suggest that *E*. *coli* L-form cells are more difficult to be cultivated in liquid medium than *Bacillus subtilis* L-form, and that L-form is in general susceptible to oxidative stress^63^.

For further examination of viability of the hybrid cells, we investigate the gene expression activity of hybrid cells from plasmid DNA molecules or mRNA molecules encapsulated in ALBiC reactors (Figs 3 and 4). In both conditions, around 20% of hybrid cells showed distinctive gene expression activity. The consistency of the populations of active hybrid cells indicates that translation is a major bottleneck of gene expression activity in hybrid cells. This point was also suggested from the comparison of hybrid cells with intact cells. The active hybrid cells produced, from over 1000 of mRNA, 20–50 molecules of β-galactosidase that corresponds to 80–100 translation events considering the homotetrameric structure of the enzyme. Intact *E*. *coli* cells produce over 10 to 1000 protein molecules from single molecule of mRNA^53^. Thus, gene expression yield of active hybrid cells is also limited by translation step. Considering the no clear correlation of activity with dilution rate, the low activity in translation step of hybrid cells might be attributable to the original viability of protoplast cells and/or the functionality of the membrane. These points remain elusive to address again the original question, ‘is it possible to rebirth a living cell after rupture?’.

One of the distinctive features of the hybrid cells is that the present method successfully incorporated a full set of components from the plasma membrane of *E*. *coli* cells (Fig. 2). Fluorescence imaging clearly proved the membrane components were fused with lipid bilayer of ALBiC reactors. We, then, attempted to measure the functionality of an incorporated membrane transporter, cytochrome *c* oxidase, by adding a water-soluble quinone. However, the nonspecific incorporation of the membrane voltage sensitive dye onto device surfaces hampered the measurement of the functionality of cytochrome *c* oxidase. The measurement of membrane functionality remained as a technical challenge for the future studies.

To demonstrate processes that are not possible or difficult using other artificial cell reactor technologies, we encapsulated and cultivated single living *E*. *coli* cells inside hybrid cells. This system can be considered as an artificial cell-in-cell system. 30% of encapsulated *E*. *coli* cells showed cell division inside of the hybrid cell reactors. Based on image analysis, entrapped *E*. *coli* cells that replicated were the same size as the daughter cells (Fig. 5a). This suggests that the cytoplasmic components of a hybrid cell contained resources for biomass production of another *E*. *coli* cell and maintained two daughter *E*. *coli* cells. However, this does not necessarily suggest that the cytoplasmic components from a single protoplast cell provided all resources to produce another *E*. *coli* cell, as hybrid cell can take up nutrient molecules from the medium to provide resources to another entrapped *E*. *coli* cell. In addition, some nutrient molecules in the culture medium outside the hybrid cells may passively diffuse across the lipid bilayer as observed for cell division in empty reactors. Recently, similar cell-in-artificial cell reactor system termed in cellular bionics system was reported where active intracellular interplay between living bacterial cell with artificial cell were programmed^40^. The present cell-in-hybrid cell system is also expected to build such cellular bionics systems.

Unexpectedly, spontaneous lysis of entrapped *E*. *coli* cells was also observed (Fig. 5b). Considering that lysis also occurred in empty reactors, it is likely that the cell lysis was caused by a physical interaction with the inner surface of the ALBiC reactor. Chemical modification of the inner wall with PEG or other biocompatible polymers may suppress this phenomenon. This finding also indicates the potential for incorporating intact genome DNA into hybrid cell reactors or fusion of hybrid cells with xenogeneic cells. Spontaneous rupture of cells in an empty reactor may enable preparation of a fresh and minimally diluted cell lysate for cell-free synthetic biology studies.

In summary, we developed a novel hybrid cell reactor composed of a single *E*. *coli* protoplast cell and artificial micron-sized reactor device. We observed gene expression activity from plasmid DNA and mRNA although clear self-replication activity was not confirmed. The viability and functionality of hybrid cells should be improved for further applications in artificial cell research. However, this system provides a novel platform for artificial cell research. As an example of an artificial parasitism system, *E*. *coli*-in-*E*. *coli* cytoplasm was prepared. Unexpectedly, cell lysis in the reactor was also observed, indicating that fresh cell extracts and intact genomic DNA can be prepared in this device.

## Methods

### Bacterial strains and construction of plasmids

In this study, strains of *E*. *coli*, Top10 (Figs 1, 3–5) and C43 (DE3) (Fig. 2) were used. To prepare *E*. *coli* cells with plasmid encoding a fluorescent protein gene with the kanamycin-resistance gene were prepared using an In-Fusion HD kit (Takara Bio, Inc., Shiga, Japan). The gene of tdTomato, mseCFP or GFPuv was respectively cloned from the ptdTomato vector (Takara Bio, Inc.), mseCFP vector (gifted from Prof. T. Nagai^64^) and the genomic DNA from an *E*. *coli* strain DH1 *ΔleuB::*(*gfpuv5-Km*^*r*^) (gifted from Prof. T. Yomo^65^). The kanamycin-resistance gene was cloned from pET-9a (Novagen, Madison, WI, USA). The fluorescent protein genes were under the control of the *lac* promoter (Figs 1, 3–5) or the T7 promoter (Fig. 2). Top10 or C43 (DE3) competent cells were transformed with these plasmids. A β-galactosidase (β-gal)-expressing plasmid was also prepared using an In-Fusion HD kit. The β-gal-coding region (*lacZ*) was cloned from *E*. *coli* BL21 (DE3). We prepared two plasmids containing *lacZ* under the *lac* promoter or *lacZ* under the T7 promoter. The former was directly introduced into the reactor, while the latter was used for *in vitro* transcription to obtain mRNA. All plasmids and strains are listed in Supplementary Table 1.

### Fabrication of micron-scaled devices

Micron-scaled devices, arrayed lipid bilayer chambers (ALBiC), were fabricated as previously described^38^. A carbon–fluorine hydrophobic polymer (CYTOP 816AP, Asahi-Glass, Tokyo, Japan) was spin-coated on a clean glass coverslip (32 × 24 mm) (Matsunami Glass Ind., Ltd., Tokyo, Japan) at 1000 rpm for 30 s and baked for 1 h at 180 °C, which resulted in a 2-μm-thick coating of CYTOP. A positive photoresist (AZP-4903, AZ Electronic Materials, Branchburg, NJ, USA) was spin-coated on the CYTOP layer at 7500 rpm for 30 s, and then baked for 3 min at 55 °C and subsequently for 5 min at 110 °C. Photolithography was carried out with a photo-mask. The resist-patterned substrate was dry-etched with O_2_ plasma using a reactive-ion etching system (RIE-10NR, Samco, Sunnyvale, CA, USA) to remove the exposed CYTOP and expose an array of hydrophilic SiO_2_ on the glass surface. The photoresist layer remaining on the substrate was removed with acetone and 2-propanol. In this study, 2 types of ALBiC were used; each diameter and depth are 4 and 2 μm (Figs 1, 3–5) or 4.5 and 3 μm (Fig. 2), respectively.

### Protoplast of *E. coli* cells

Protoplasts were prepared as previously described^39^ with some modifications. *Escherichia coli* were grown in Luria-Bertani (LB) medium containing 50 μg/mL of kanamycin (Wako, Osaka, Japan) until the growth reached the exponential phase (OD_600_ = 0.6). Then, the *E*. *coli* cells were harvested by centrifugation at 3500 × *g* for 10 min at 30 °C. The cell pellet was suspended in 3 mL of SP buffer (25 mM Tris-HCl, pH 7.4, and 300 mM sucrose). Lysozyme (Wako) (60 mg/mL) and 0.7 U/mL of DNase I (Takara Bio, Inc.) were added to the cell suspension followed by incubation for 15 min at 30 °C. The *E*. *coli* cells were harvested and resuspended in 20 mL of GP medium (2.75% trypticase soy broth without dextrose (BD Biosciences, San Jose, CA, USA), 10 mM MgSO_4_, 50 mM KCl, and 300 mM sucrose), containing ampicillin (Wako) (800 μg/mL) and kanamycin (50 μg/mL). The protoplast suspension in GP medium was incubated by shaking at 30 °C for 3–4 h at 30 rpm. As incubation time increased, protoplast size also increased. Protoplasts were harvested by centrifugation at 2300 × *g* for 15 min at 30 °C. Prepared protoplast cells were 2.0 ± 0.48 μm in diameter.

### Image acquisition and data analysis

Time-lapse recordings of fluorescent images were taken under a confocal microscope system (A1, Nikon, Tokyo, Japan). Three types of lasers with the wavelength of 400 nm (mseCFP, Alexa405), 488 nm (Alexa488, TopFluor-PS, GFPuv, SPiDER-βGal), or 561 nm (tdTomato), were used. Images were acquired with a ×60 objective lens and photomultiplier tubes with corresponding detectors (mseCFP and Alexa405 425–475 nm; Alexa488, TopFluor-PS, GFPuv and SPiDER-βGal 500–550 nm; tdTomato 570–620 nm). Image analysis was performed using NIS Elements software (Nikon).

### Preparation of flow channel and hybrid cells

Flow cells, each having two flow channels assembled from ALBiC device and a top cover glass, were prepared as previously described^38^. To form lipid bilayer membranes on the ALBiC reactors, media were sequentially introduced into a flow channel. First, 20 μL of SP buffer containing 5 μM Alexa Fluor 488 (Alexa488) (Thermo Fischer Scientific, Waltham, MA, USA) or Alexa Fluor 405 (Alexa405) (Thermo Fischer Scientific) was injected into a flow channel. Next, 20 μL of lipid solution containing 1.0 mg/mL 1,2-dioleoyl-*sn*-glycero-3-phosphocholine (DOPC) (Avanti Polar Lipids, Alabaster, AL, USA) and 0.2 mg/mL 1,2-dioleoyl-*sn*-glycero-3-phosphoethanolamine (DOPE) (Avanti Polar Lipids) in hexadecane (Sigma-Aldrich, St. Louis, MO, USA) was injected to flush away the first SP buffer. The components of lipid molecules were decided by the pilot experiment. Although using *E*. *coli* total lipids would be more suitable for the viability of the hybrid cell, the reproducibility of the hybrid cell was not high in this condition. Next, GP medium was injected as an aqueous solution to flush away the lipid solution. The residual lipids molecules formed a lipid membrane on each reactor. For the preparation of hybrid cells, the suspension of protoplast cells was injected into the flow channel with kanamycin (50 μg/mL). After 30–90 min incubation, the suspension was washed out to eliminate non-fused protoplasts for clarity of the observation. Hybrid cells were identified as ALBiC reactors showing fluorescence signal of protoplasts as described in the main text.

### Imaging of plasma membrane fusion of protoplast

To visualize the fusion of protoplast membrane with the lipid bilayer of ALBiC, the plasma membrane of protoplasts was fluorescently labeled as follows. Protoplasts of C43 (DE3) cells expressing tdTomato were prepared as described above. The expression level of tdTomato was suppressed compared with experiments in Fig. 1 to avoid the fluorescence crosstalk with TopFluor. They were harvested by centrifugation at 2300 × *g* for 15 min at 30 °C to remove the culture medium, carefully washed twice with SP2 buffer (SP buffer containing 50 mM KCl and 50 mM MgCl_2_) and finally resuspended with SP2 buffer. Fluorescent-labeled lipid 1-palmitoyl-2-(dipyrrometheneboron difluoride)undecanoyl-*sn*-glycero-3-phospho-L-serine (TopFluor-PS, Avanti Polar Lipids) dissolved in chloroform was dried and resuspended in DMSO with the concentration of 1 mg/mL. DMSO-solubilized TopFluor-PS was added to the protoplasts solution with a final concentration of 0.002 mg/mL. After 1 hour incubation at 30 °C, the protoplasts solution was diluted 50-fold with GP buffer and injected to a flow cell. For the visualization of the integrity of ALBiC membrane, the inner medium of ALBiC was stained with Alexa405 in SP buffer. Fluorescent images were obtained in the multipoint time-lapse measurement.

### β-Gal expression in hybrid cells

To measure transcription and translation activities of hybrid cells, plasmid DNA or mRNA that encodes *lacZ* was introduced into ALBiC reactors prior to fusion with protoplast cells. mRNA encoding *lacZ* was prepared by using *in vitro* Transcription T7 Kit (Takara Bio, Inc.), and was purified using the NucleoSpin RNA Clean-up XS (Takara Bio, Inc.). The yield of the transcripts was measured by a NanoDrop 2000 Spectrophotometer (Thermo Fischer Scientific). The transcripts were freshly prepared before each assay. *E*. *coli* strain Top10 expressing mseCFP was used for protoplast preparation, as *lacZ* genes are knocked out in Top10. SP buffer containing 30 ng/μL of plasmid or 95 ng/μL of mRNA and 5 mM of ATP were introduced in the ALBiC reactors before fusion, resulting in approximately 2.73 × 10^2^ molecules of plasmid or 1.44 × 10^3^ molecules of mRNA in each reactor. After 3 h of incubation, 150 μL of GP medium containing 100 μM spiro-based immobilisable diethylrhodol-βGal (SPiDER-βGal)^52^ was injected into the ALBiC.

### Estimation of the number of expressed β-Gal molecules

To estimate how many β-gal molecules were expressed in hybrid cells, the calibration line of fluorescence increment (AU/h) was determined as the function of the number of β-gal molecules in ALBiC reactors (Supplementary Fig. 2). Purified β-gal (Wako) was diluted in SP buffer to 0.44, 0.88, and 1.76 nM to achieve 5, 10, and 20 molecules per reactor, respectively. The fluorescence increment was determined from the 3-hour time course after injecting SPiDER-βGal.

### Calculation of dilution rate via fusion

We investigated the correlation between gene expression activity and the dilution rate of the protoplast cytosol upon fusion (Supplementary Fig. 3). The dilution rate was determined from the intensity decrease of Alexa405, which was added to the inner solution of the ALBiC reactors before fusion. The volume of a reactor, protoplast, and hybrid cell are indicated as *V*_*reactor*_, *V*_*protoplast*_, and *V*_*hybrid cell*_, respectively. Since the amount of Alexa405 in each reactor was constant, even after protoplast fusion, the ratio of Alexa405 intensity after fusion to before fusion (*Int*_*after*_/*Int*_*before*_) is described below:

1 $$\frac{In{t}_{after}}{In{t}_{before}}=\frac{{V}_{reactor}}{{V}_{reactor}+{V}_{protoplast}}$$

Therefore, the dilution rate, which is the volume ratio of a hybrid cell to a protoplast (*V*_*hybrid cell*_/*V*_*protoplast*_), is described as follows:

2 $$\frac{{V}_{hybridcell}}{{V}_{protoplast}}=\frac{1}{\frac{{V}_{protoplast}}{{V}_{reactor}+{V}_{protoplast}}}=\frac{1}{1-\frac{In{t}_{after}}{In{t}_{before}}}$$

The β-gal expression experiment was conducted as described with the following modifications. The *E*. *coli* strain used was Top10 expressing GFPuv rather than mseCFP because of the similarity of the florescent spectra between mseCFP and Alexa405. After injecting SP buffer containing 100 μM of SPiDER-βGal, time-lapse recording of the intensity of Alexa405 and SPiDER-βGal was simultaneously conducted. The fluorescent intensity of SPiDER-βGal was recorded at 570–620 nm to avoid fluorescent crosstalk with the emission spectrum of GFPuv. The slope value of the SPiDER-βGal intensity change and dilution rate of each hybrid cell was calculated and shown as a histogram (Supplementary Fig. 3). We calculated *Int*_*before*_ as fluorescence intensity of nearby reactors of the fused reactor, as it is not possible to know which reactors fused to a protoplast cell in advance.

### *E. coli* in hybrid cell

To introduce normal *E*. *coli* cells into a hybrid cell, *E*. *coli* expressing tdTomato were cultured in LB medium containing kanamycin. In the exponential phase, cells were harvested by centrifugation and resuspended in SP buffer. The OD value was measured, and the cells were diluted to OD = 0.2. *E*. *coli* cells were entrapped in hybrid cells, as same as plasmid or mRNA entrapment.

## Electronic supplementary material

Supplementary Information

Supplementary movie 1

Supplementary movie 2
