# Supplementary material for: Hybrid cell reactor system from Escherichia coli protoplast cells and arrayed lipid bilayer chamber device
Source: Sci Rep. 2018 Aug 6;8:11757. doi: 10.1038/s41598-018-30231-0 (PMC6078950; doi:10.1038/s41598-018-30231-0)
Supplement: Supplementary file 1 — Supplementary Information [file 41598_2018_30231_MOESM1_ESM.docx]

**Supplementary Information for**

Hybrid cell reactor system from *Escherichia coli* protoplast cells and arrayed lipid bilayer chamber device

#^1^ Yoshiki Moriizumi, *#^1, 4, 7^ Kazuhito V. Tabata, ^1, 4, 5^ Rikiya Watanabe, ^2^ Tomohiro Doura, ^2, 4^ Mako Kamiya, ^2, 3, 6^ Yasuteru Urano, *^1, 7^ Hiroyuki Noji.

# contributed equally

* Corresponding authors

^1^Department of Applied Chemistry, Graduate School of Engineering, The University of Tokyo, 7-3-1, Hongo, Bunkyo-ku, Tokyo 113-8656, Japan

^2^Graduate School of Medicine, The University of Tokyo, 7-3-1, Hongo, Bunkyo-ku, Tokyo 113-0033, Japan

^3^Graduate School of Pharmaceutical Sciences, The University of Tokyo, 7-3-1, Hongo, Bunkyo-ku, Tokyo 113-0033, Japan

^4^PRESTO, Japan Science and Technology Agency, 4-1-8, Honcho, Kawaguchi, Saitama 332-0012, Japan

^5^PRIME, Agency for Medical Research and Development, 1-7-1, Otemachi, Chiyoda-ku, Tokyo 100-0004, Japan

^6^CREST, Agency for Medical Research and Development, 1-7-1, Otemachi, Chiyoda-ku, Tokyo 100-0004, Japan

^7^ImPACT, Council for Science, Technology and Innovation, Cabinet office, Government of Japan, 1-6-1, Nagata-cho, Chiyoda-ku, Tokyo 100-8914, Japan

**Supplementary Table 1.** *E. coli* strains and phenotypes used for this study.

| ***E. coli* strains used for this study** | |  |  |  |
| --- | --- | --- | --- | --- |
| **Experiment** | **Purpose** | ***E. coli* strain** | **Fluorescent protein (color)** | **Promoter for protein expression** |
| Figure 1 | Protoplast for fusion | Top10 | tdTomato (red) | *lac* |
| Figure 2 | Protoplast for fusion | C43 (DE3) | tdTomato (red) | *T7* |
| Figure 3 | Protoplast for fusion | Top10 | mseCFP (cyan) | *lac* |
| Figure 4 | Protoplast for fusion | Top10 | mseCFP (cyan) | *lac* |
| Figure 5 | Protoplast for fusion | Top10 | GFPuv (green) | *lac* |
|  | Encapsulate in the reactor | Top10 | tdTomato (red) | *lac* |
| Supplementary Figure 3 | Protoplast for fusion | Top10 | GFPuv (green) | *lac* |
|  |  |  |  |  |
| **Genotype of the *E. coli* strains** | |  |  |  |
| Top10 | F^-^ *mcr*A Δ(*mrr-hsd*RMS-*mcr*BC) φ80*lac*ZΔM15 Δ*lac*X74 *rec*A1 *ara*D139 Δ(*ara-leu*)7697 *gal*U *gal*K *rps*L (Str^R^) *end*A1 *nup*G | | | |
| C43 (DE3) | F^-^ *ompT hsdSB (rB- mB-) gal dcm*(DE3) | | | |

**
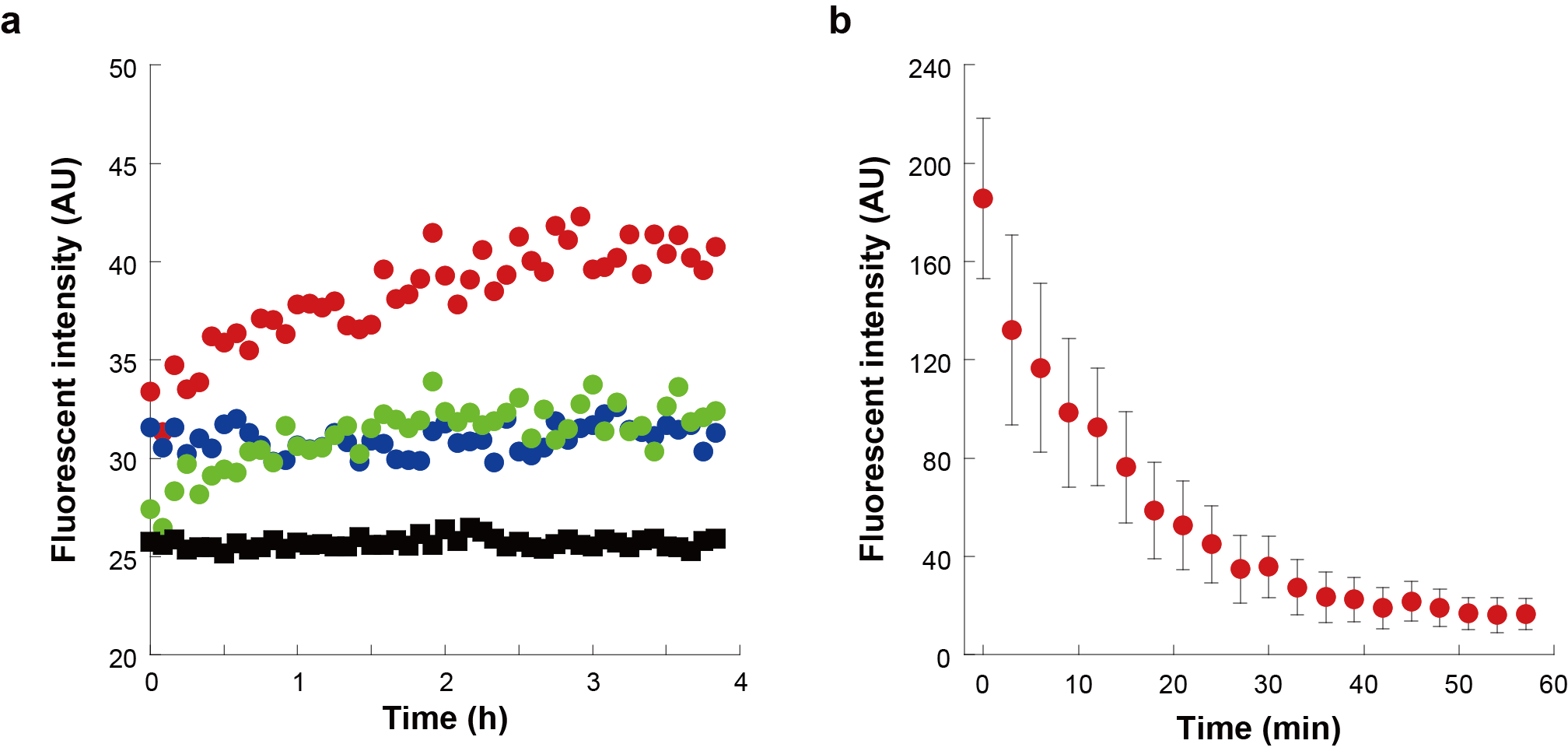
**

**Supplementary Figure 1.** β-Gal expression assay from inserted mRNA using fluorescein di-β-D-galactopyranoside (FDG).

(a) Time courses of FDG fluorescence changes in hybrid cell. The assay was conducted as described in Fig. 4 except that 7 μM of FDG was added rather than SPiDER-βGal. In this experiment, red, green, and blue dots are examples of time courses, and black dots show the time course of the negative experiment without mRNA.

(b) Time course of fluorescence decay of fluorescein encapsulated in ALBiC reactors. Time-lapse observation was immediately followed by encapsulating 10 μM of fluorescein, which is a product of FDG, into the reactors. Decay of intensity demonstrated the membrane permeability of fluorescein. Error bars indicate s.d.


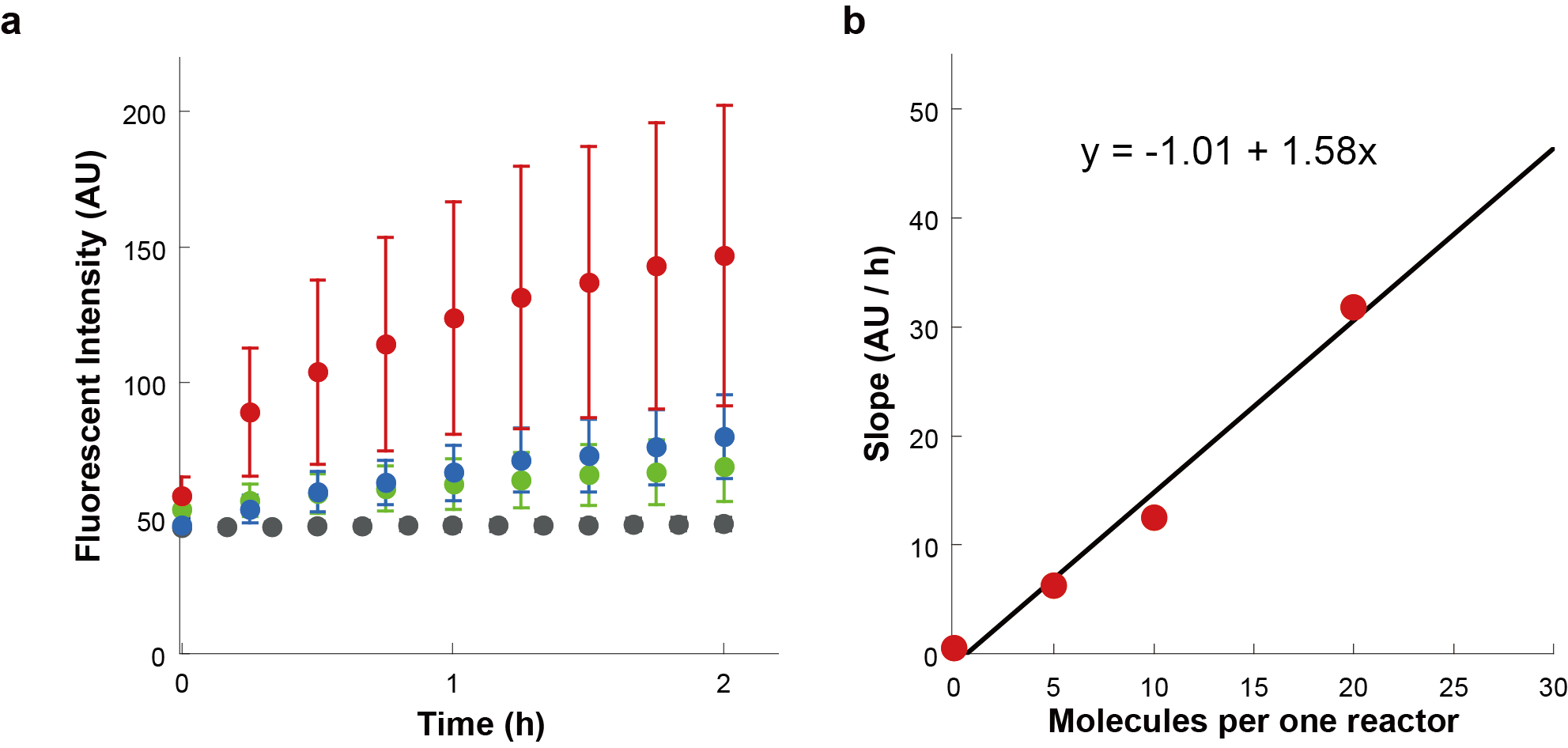


**Supplementary Figure 2.** Relationship between time course of SPiDER-βGal^1^ fluorescence change and number of β-gal molecules in a hybrid cell.

(a) Time courses of SPiDER-βGal fluorescence change with various β-gal molecule numbers encapsulated in each hybrid cell. Red, blue, green and gray dots indicate 20, 10, 5 and 0 molecules, respectively.

(b) Plot showing relationship between slope (AU/h) of the time courses and β-gal molecule number in the hybrid cell. Each dot indicates the average of slope values in several tens of hybrid cells. Lines are linearly fitted. At higher molecular numbers, many β-gal were released from membrane-broken reactors and hydrolyzed SPiDER-βGal, and thus the aqueous solution in the flow channel showed a fluorescence intensity that was too high for observation of hybrid cells.


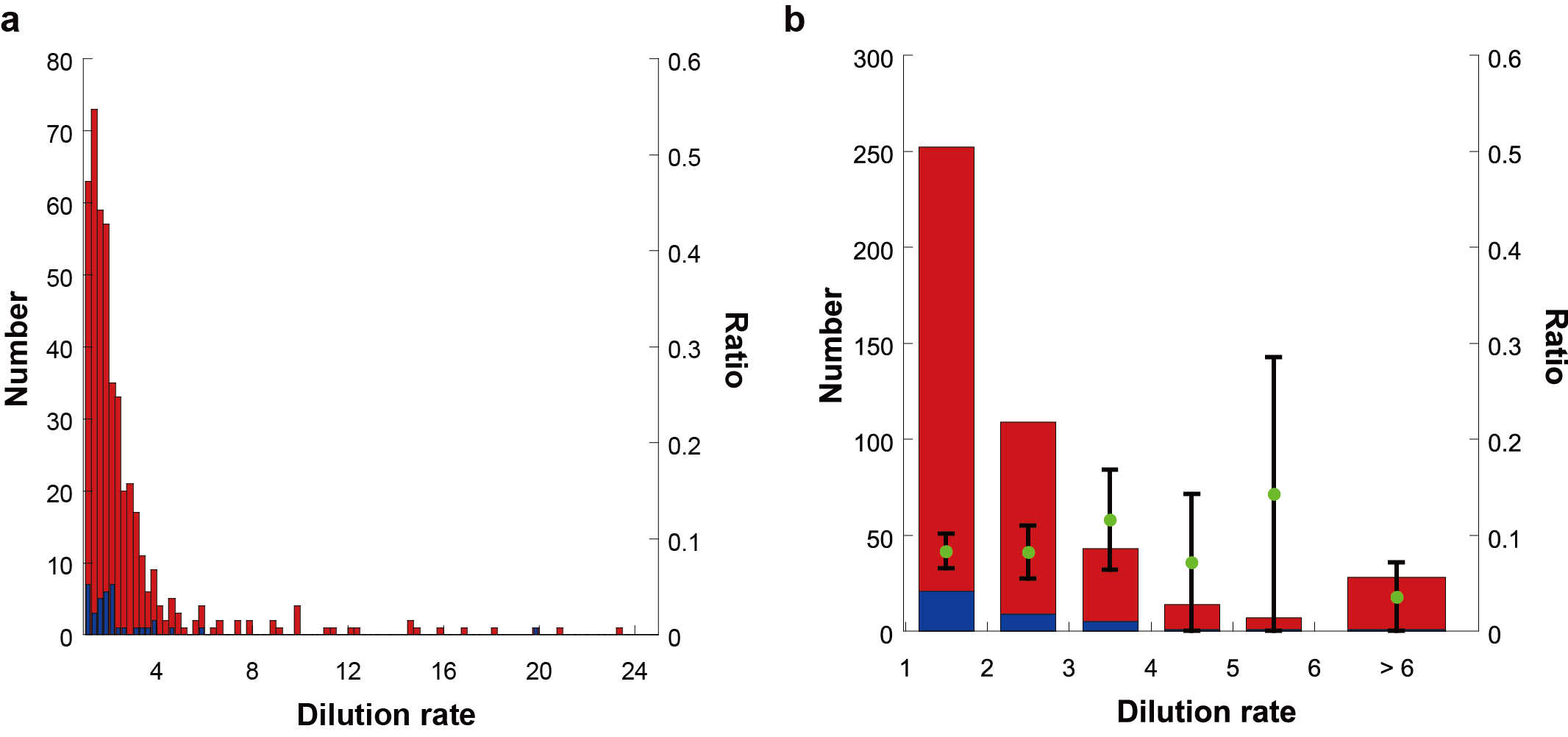


**Supplementary Figure 3.** Dilution rate versus gene expression activity.

(a) Histogram of the numbers of total hybrid cells (red) and active hybrid cells for gene expression (blue) with different dilution rate. Dilution rate of each hybrid cells was calculated from the intensity decrease of Alexa405 encapsulated in the ALBiC reactors before fusion (see also *Methods*).

(b) Enlarged histogram of (a). Green data points represent the ratio of the number of active hybrid cells to total cells. Error bars indicate s.d.

**Supplementary Movie 1–2.** Morphological change of hybrid cell membrane.

Bright-field movies and GFPuv fluorescent movies were merged. Note that these movies showed the full length of the experiment (not only the moment when protrusion occurred). Images were recorded every 6 min for more than 10 h. The frame rate of these movie is 10 frames per s. Scale bars indicate 5 μm.

**Supplementary Reference**

1. Doura, T. *et al.* Detection of LacZ-Positive Cells in Living Tissue with Single-Cell Resolution. *Angew. Chemie - Int. Ed.* **55,** 9620–9624 (2016).
